# Evaluation of Anxiolytic, Sedative-hypnotic and Amnesic Effects of Novel 2-phenoxy phenyl-1,3,4-oxadizole Derivatives Using Experimental Models

**Published:** 2015

**Authors:** Sayyed Abbas Tabatabai, Elham Rezaee Zavareh, Hamed Reyhanfard, Bagher Alinezhad, Bijan Shafaghi, Majid Sheikhha, Abbas Shafiee, Mehrdad Faizi

**Affiliations:** a *Department of Pharmaceutical Chemistry, School of Pharmacy, Shahid Beheshti University of Medical Sciences, Tehran, Iran.*; b *Department of Pharmacology and Toxicology, School of Pharmacy, Shahid Beheshti University of Medical Sciences, Tehran, Iran.*; c *Department of Medicinal Chemistry, School of Pharmacy, Tehran University of Medical Sciences, Tehran, Iran.*

**Keywords:** 1, 3, 4-oxadiazole, Hypnotic, Anxiolytic, Amnesic

## Abstract

Benzodiazepines (BZDs) are widely used in clinical practice as anxiolytics, hypnotics, anticonvulsants and muscle relaxants. However, they have some undesired effects including memory problems. In continuing our research on novel benzodiazepine ligands, we are looking for ligands with less adverse effects. Previously, 4 novel derivatives of 2-phenoxy phenyl-1,3,4-oxadiazole were synthesized as agonists of BZD receptors. In this study, the pharmacological effects of novel compounds were evaluated. Pentobarbital induced loss of righting reflex, elevated plus maze, open-field locomotor activity and passive avoidance test were used to evaluate the sedative-hypnotic, anxiolytic and amnesic effects of compounds respectively. The results revealed that the novel compounds with NH_2_, SH and SCH_3_ substituents at the 2-position of the oxadiazole ring increase righting reflex time significantly. In the elevated plus maze test none of the derivatives increased open arm duration and open arm entry indicating no anxiolytic properties. Moreover, the novel compounds didn’t influence step-down latencies in the mice. The fact that the hypnotic activity of these compounds were significantly reduced by flumazenil, confirmed that this effect is mediated by BZD receptors.

## Introduction

Agonists of benzodiazepine (BZD) binding site in GABA_A_ receptors are important classes of drugs used in the treatment or control of some central nervous system (CNS) diseases (1). They act by improving the gamma-aminobutyric acid (GABA_A_) receptor function in the central nervous system. Agonists of BZD receptors increase the frequency of the opening of the chlorine channel in response to GABA action which cause anticonvulsant, anxiolytic, sedative and muscle relaxant effects (2-6). In spite of their quick onset of action and low toxicity, benzodiazepines have some undesirable effects such as sedation, negative effect on memory, and development of tolerance to the desirable effects (7). Therefore, the need for novel agonists of benzodiazepine receptors with different chemical and pharmacological structures has been the recent lookout for effective CNS therapy. Our previous studies on BZD receptor ligands showed that some simple non-rigid derivatives with five membered heterocycle rings such as triazoles, oxadiazoles and thiadiazoles had appropriate pharmacological effects (8-15*)*. As shown in Table 1, the Novel 1,3,4-Oxadiazole derivatives were synthetized in order to have selective anticonvulsant or sedative effects with less undesired effects including memory problems (16). In the present study, as an *in-vivo* model for evaluating BZD effects, elevated plus-maze (EPM), step down passive avoidance and righting reflex tests were performed on novel 2-[2-(2-Chlorophenoxy) phenyl]-1,3,4-oxadiazole derivatives as candidates for agonistic effect on benzodiazepine receptors. To confirm the mode of action of novel compounds, the effect of flumazenil, a BZD receptor antagonist, on the anticonvulsant activity of the compounds has been reported.

**Table 1:** Chemical structures of the novel 2-phenoxy phenyl-1,3,4-oxadizole derivatives.

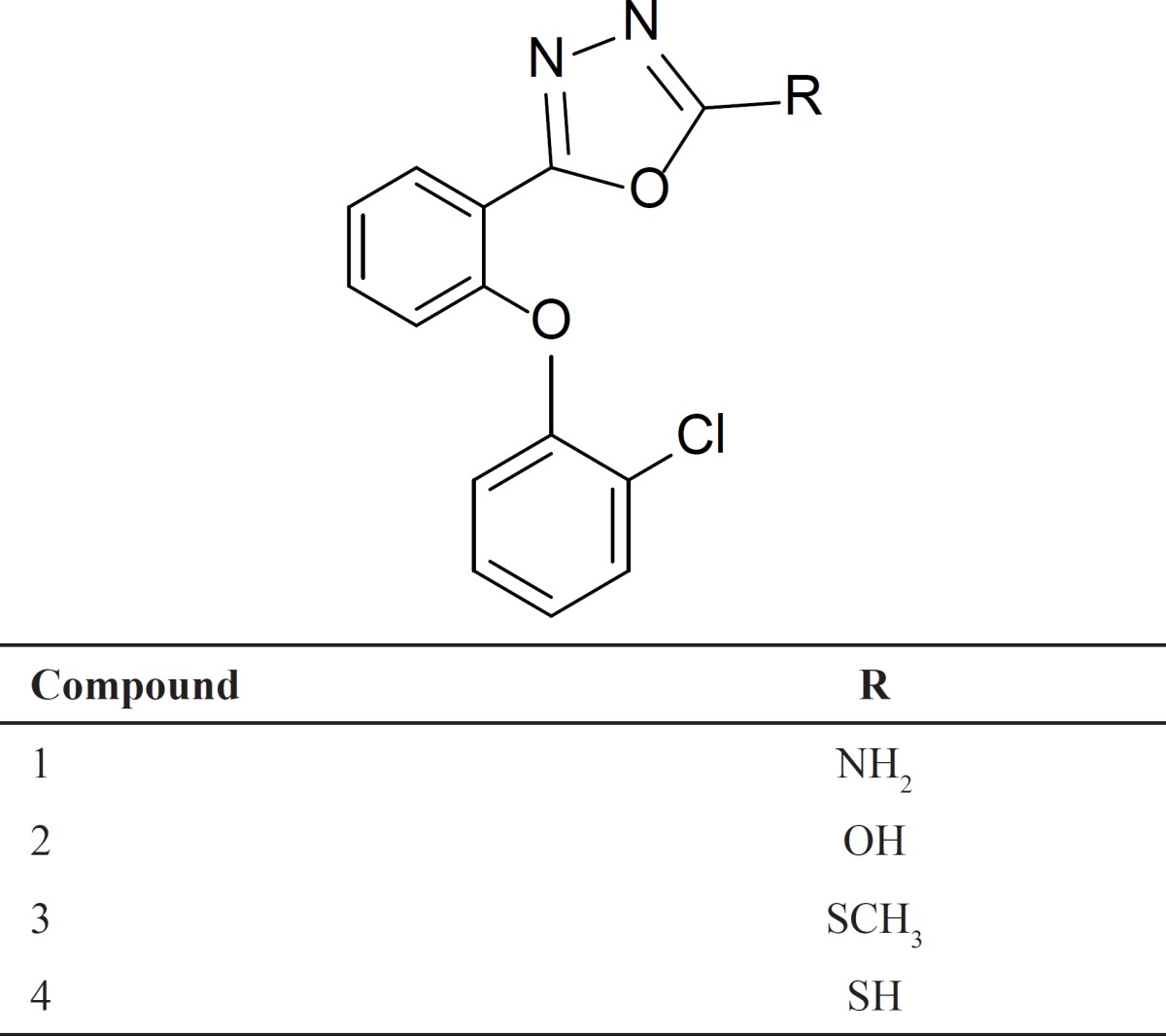

## Experimental

*Materials and methods*

*Animals*

The animals used for the experiments were male NMRI mice (Pasteur Institute, Iran) weighting in the range of 20-25 g. The animals were housed in a controlled condition and 12 h light/dark cycle. All pharmacological experiments were performed between 9:00 and 15:00. Standard mouse diet and water were freely available to them except during the experiment. Thirty minutes before the experiment, the animals were selected randomly and transferred to individual cages and allowed to acclimatize before injecting drugs or vehicle. This study was performed in accordance with a set of rules approved by the institutional animal care and use committee. All experiments were conducted based on the National Institutes of Health (NIH) Guide for the Care and Use of Laboratory Animals and all efforts were made to minimize the number of animals used in the study.

*Compounds*

As previously described, the novel compounds were synthetized in Shahid Beheshti University of Medical Sciences (16). Flumazenil (Haffman La Rosche), diazepam (Sigma) and sodium pentobarbital (Sigma) were given intraperitoneally (*i.p*.) as freshly prepared solutions. The novel compounds diazepam and flumazenil were used in a suspension of 1% CMC and 0.5% Tween 80. Pentobarbital was dissolved in water. The injection volume was 10 mL/Kg and the control group was injected with a suspension of 1% CMC and 0.5% Tween 80 (vehicle).

*Elevated plus-maze (EPM) test*

The elevated plus maze apparatus was made up of Plexiglas and it was consisted of two open arms (30×5×0.5 cm) and two closed arms (30×5×35 cm ) with an open roof. They were arranged so that the open arms were opposite to each other and all the four arms were connected together with a central square of 5×5 cm. The entire maze was elevated at a height of 1 m and was placed inside a light and sound attenuated room (17, 18-20). The mice were placed in the center of the maze facing towards one of the closed arms 30 minutes after intraperitoneal (*i.p*) administration of test drug or diazepam as standard drug. The number of entries and also the time spent in the open arm were recorded (17). The values for the test compound treated groups were compared with that of the vehicle treated and the standard drug treated groups. In this test, three major parameters were measured including the number of entries in the open and closed arms and the time spent in the open arm of the EPM during a 5 min test session.

*Righting reflex test*

Righting reflex test was used for the evaluation of hypnotic effect. The test is based on potentiating the pentobarbital induced sleeping time (loss of righting reflex). Thirty min after the administration of novel compounds or diazepam, pentobarbital (40 mg/Kg, *ip*) was given to induce sleep (21-22). The interval between the loss and recovery of reflex was used as an index of hypnotic effect. The time interval between the injection of pentobarbital and start of losing righting reflex was recorded as latency time. For the compounds with significant hypnotic effects, flumazenil was used to investigate the involvement of benzodiazepine receptors in hypnotic effect of the compounds. Flumazenil was given (10 mg/Kg, *i.p*.) 15 min before injection of diazepam or novel compounds. Then, the righting reflex test was performed after 30 min.

*Step-down passive avoidance test*

In the present study, a modification of step-down passive avoidance test was used to assess learning and memory in mice (22). The apparatus (Malek Teb Co., Tehran) consisted of a grid floor with a wooden block (4×4×4 cm) was placed in the center. The block served as a shock free zone. For training trial, each mouse was placed on the wooden platform set in the center of the grid floor. When the mouse stepped down and placed all four paws on the grid floor, the electric shock (0.5 mA, 1 Hz) was given through the grid floor on stepping down. Shocks were delivered for 16 s and the step- down latency (SDL) was recorded. SDL was defined as the time taken by the mouse to step down from wooden platform to grid floor with its entire paw on the grid floor. Mice which showed SDL in the range (2–16 s) during the first test were used for the second session and the retention test (22). Retention test was conducted 24 h later without shock. The time taken for the animal to step down was recorded as step-down latency as a measure of retention. A cut-off time of 300 s was chosen. Novel compounds and diazepam were administered 60 min prior to the training trial. Prolongation of step-down latency was used as a parameter of learning.

*Statistical analysis*

Statistical analysis of the anxiolytic activity of novel compounds on animals was evaluated using a one-way analysis of variance (ANOVA). In all cases, post-hoc comparisons of the means of individual groups were performed using a Fisher’s Exact Probability test. All values were expressed as mean ± SD (standard deviations). Graph pad Prism software version 5.04 was used for statistical analysis and *p *< 0.05 was considered statistically significant. Probit-regression method and SPSS software (Chicago, IL; version 13) were used to determine all ED_50 _values.

## Results

*Elevated-plus maze test*

In the elevated plus maze, the frequency of entries onto the open and closed arms was noted and the time spent on the open arms was measured over 5 minutes. The minimum number of mice in each dose was twelve. In this test, entry onto either arm was counted when the mouse had its body and four paws on the arm. As shown in Table 2, diazepam showed a significant increase in the OAT (open arm time), OAE (open arm entries), TAE (total arm entries) and no effect in the CAE (closed arm entries). There was no significant effect for compounds 1, 2 and 4. Compound 3 had a significant increase on OAE (*p *< 0.05), CAE (*p *<0.01) and TAE (*p *<0.01) which indicated an increase on locomotor activity. Test compounds didn’t show any prominent effect on the time spent on the open arm (OAT).

**Table 2:** The results of the elevated-plus maze test on diazepam and the novel 2-phenoxy phenyl-1,3,4-oxadizole derivatives.

**Compound**	**Dose (mg/Kg)**	**OAT** a ** (s)**	**OAE** b ** (n)**	**CAE** c ** (n)**	**TAE** d ** (n)**	**Estimated potency (mg/Kg) Mean ± SEM**
1	10	62.66 ± 23.50	2.75 ± 0.52	4.75 ± 1.12	7.50 ± 1.41	ND^e^
	20	23.50±26.97	1.16± 0.42	2.16±0.47	3.33± 0.81	
	30	66.08±29.40	1.25± 0.57	3.00± 0.93	4.25± 1.14	
	50	73.16±25.55	1.58± 0.39	1.83± 0.32	3.41± 0.55	
2	10	48.33±12.71	3.16± 0.53	5.85± 0.85	5.58± 0.85	ND
	20	27.91± 8.45	1.83± 0.71	4.08± 0.83	4.08± 0.85	
	30	61.25± 28.7	1.25± 0.41	1.83± 0.57	1.83±0.57	
	50	92.83± 29.55	2.75± 0.67	4.5± 1.07	4.58± 1.07	
3	30	92.08± 9.40	6.66± 1.38 *	10.50± 1.46 **	17.25± 2.62**	ND
	50	92.41± 26.81	4.08± 1.01	6.08± 1.22	10.16± 2.1	
4	30	58.33± 8.58	3.83± 0.51	6.5± 0.91	10.33± 7.56	ND
	50	82.50± 19.50	3.75± 0.73	6.75± 1.04	10.50± 7.02	
Diazepam	vehicle	54.00 ±25.03	2.0 ± 0.82	4.33 ± 0.93	6.33 ±1.39	0.836 ± 0.130
	0.5	63.75 ± 15.85	4.5 ± 1.58	5.66 ± 1.30	10.16 ± 2.7	
	1	123.66 ± 25.94*	4.91 ± 1.35	4.75 ± 1.31	9.66 ± 2.37	
	2	182.41 ± 24.51***	9.41 ± 1.88*	4.5 ± 1.15	13.9 ± 1.71*	

a Open arm time.

b Open arm entries.

c Closed arm entries.

d Total arm entries.

e Not determined because there was no effect in all EPM parameters.

* *p *< 0.05, significant difference compared with the control group (n = 12).

*** *p* < 0.001, significant difference compared with the control group (n = 12).

*Righting reflex test*

As shown in Table 3, except compound 2, all compounds significantly increased the time of righting test. ED_50 _was measured for all compounds. The duration of the loss of righting reflex was assessed. The minimum number of mice in each group was twelve. As shown in Table 3, there is a significant difference between diazepam and test compounds. 

**Table 3:** Effects of diazepam and the novel 2-phenoxy phenyl-1,3,4-oxadizole derivatives on loss of righting reflex.

**Compound**	**Dose (mg/Kg)**	**Response (s)** **Mean± SEM**	**ED**_50_ ** (mg/Kg) Mean ± SEM**	**ED**_50_ ** (mg/Kg) Mean ± SEM (in the presence of flumazenil)**	**p-value** a
1	Vehicle	814.17 ± 51.793	14.927 **± **1.100	68.960 ± 1.069	*p* < 0.001
	5	946.67 ± 124.570			
	10	1321.67 ± 101.699*			
	20	1650.00 ± 102.956***			
	30	2108.33 ± 217.062***			
	50	2615.00 ± 169.563***			
2	Vehicle	814.17 ± 51.793	ND^b^	ND	*p* > 0.05
	5	830.00 ± 104.849			
	10	931.67 ± 89.309			
	20	868.33 ± 109.527			
	30	878.33 ± 71.620			
	50	1117.5 ± 65.41			
3	Vehicle	814.17 ± 51.793	20.606 **± **1.113	54.074 ± 1.095	*p* < 0.001
	5	798.33 ± 85.222			
	10	1183.33 ± 81.921			
	20	1795.00 ± 157.792***			
	30	1613.33 ± 101.314***			
	50	2225.00 ± 190.416***			
4	Vehicle	814.17 ± 51.793	17.338 **± **1.108	44.055 ± 1.098	*p* < 0.001
	5	905.00 ± 77.233			
	10	1321.67 ± 88.823*			
	20	1891.67 ± 144.347**			
	30	1710.00 ± 150.244*			
	50	2288.33 ± 137.366***			
Diazepam	Vehicle	814.17 ± 51.793	0.462 **± **1.115	1.430 ± 1.112	*p* < 0.001
	0.5	1705.83 ± 93.812***			
	1.25	2654.17 ± 103.554***			
	2.5	3345.00 ± 112.509***			

a p-values: significance level for deviation of slope from zero.

b Not determined because there was no significant hypnotic effect.

* *p* < 0.05, significant difference compared with the vehicle group (n = 12).

** *p* < 0.01, significant difference compared with the vehicle group (n = 12).

*** *p* < 0.001, significant difference compared with the vehicle group (n = 12).

*Step-down passive avoidance test*

To evaluate the effects of the novel compounds on memory, prolongation of step-down latency was used as a parameter of learning and memory. Results of the step-down passive avoidance test have been shown in Table 4. The results show that none of the novel compounds change the step down latency significantly.

**Table 4:** Effects of diazepam and the novel 2-phenoxy phenyl-1,3,4-oxadizole derivatives on step-down latency.

**Compound**	**Dose (mg/Kg)**	**Response (s) Mean ± SEM**	**Estimated potency (mg/Kg) Mean ± SEM**	**p-value** a
1	Vehicle	231.12 ± 10.79	ND^b^	*p* > 0.05
	25	233.50 ± 16.35		
	50	227.37 ± 23.44		
2	Vehicle	231.12 ± 10.79	ND	*p* > 0.05
	25	244.75 ± 13.93		
	50	216.62 ± 19.84		
3	Vehicle	231.12 ± 10.79	ND	*p* > 0.05
	25	236.12 ± 16.96		
	50	193.75 ± 31.12		
4	Vehicle	231.12 ± 10.79	ND	*p* > 0.05
	25	225.87 ± 18.58		
	50	207.25 ± 21.18		
Diazepam	Vehicle	231.12 ± 10.79	3.041 ± 1.479	*p* < 0.001
	1	175.37 ± 12.88**		
	2	136.62 ± 13.03***		

a p-values: significance level for deviation of slope from zero.

b Not determined because there was no significant effect on memory.

** *p* < 0.01, significant difference compared with the vehicle group (n = 12).

*** *p* < 0.001, significant difference compared with the vehicle group (n = 12).

## Discussion

An effective anxiolytic agent should reduce anxiety and exert a calming effect. On the other hand, a hypnotic drug should produce drowsiness and encourage the onset and maintenance of a state of sleep. Hypnotic effects involve more pronounced depression of the central nervous system than sedation and this can be achieved with many BZDs. Many of the common adverse effects of hypnotic agents result from dose-related depression of the central nervous system. Relatively low doses of BZDs may lead to drowsiness, impaired judgment and diminished motor skills. Aside from their quick onset of action and low toxicity, benzodiazepines have some undesirable effects such as sedation, negative effect on cognition, and development of tolerance to the desirable effects. Therefore, synthesis of novel agonists of benzodiazepine receptors with different chemical structure is still an important challenge. In this study, pharmacological evaluation of anxiolytic, sedative-hypnotic and memory impairment effects of the novel compounds and diazepam, as a reference, were tested using three well-known tests (23). Results of righting reflex test clearly indicated that the compounds with NH_2_, SH, or SCH_3 _groups on 2-position of 1,3,4-oxadiazole ring have a considerable hypnotic effect. However, their potencies were less than diazepam and there was no significant difference among those of the three compounds. It means the compounds with NH_2_, SCH_3_, or SH groups have similar hypnotic effects and compound with OH group on 2-position of the heterocyclic ring did not show hypnotic effect. The hypnotic activities of three compounds as well as diazepam were reduced by flumazenil; it concludes that these effects were mediated through benzodiazepine receptors. All the compounds show no significant effects on memory and anxiety in step-down passive avoidance and elevated-plus maze test respectively. Since the previously reported study showed that the compound with OH substituents on 2-position of 1,3,4- oxadiazole ring (Compound 2) has a considerable anticonvulsant activity (16), Compound 2 might be a valuable lead compound to develop novel potent anticonvulsant agents with no impairment on learning and memory. The observed results in this study are completely compatible with our previous studies on other 1,3,4-oxadiazole and 1,2,4-triazole derivatives. In all investigated heterocyclic derivatives which were introduced as benzodiazepine receptor ligands, the amino substituent at the same position had the best effect on both hypnotic and anticonvulsant activities beside no effect on memory (8-15). The pharmacological relevance of the multitude of structurally diverse GABA_A_ receptor subtypes determines that α_1_ subunit of the GABA_A_ receptors is responsible for hypnotic activity of BZD agonists and also the effect on memory is mediated through the GABA_A_ receptors with α_5 _subunit (24). Since novel compounds had hypnotic activity with no effect on memory; it seems that these compounds may have higher affinity for α_1 _than α_5 _subunit. However, further studies are needed to prove this hypothesis. 
